# High Risk Stage 2 and Stage 3 Colon Cancer, Predictors of Recurrence and Effect of Adjuvant Therapy in a Nonselected Population

**DOI:** 10.1155/2015/790186

**Published:** 2015-06-21

**Authors:** Elmer E. van Eeghen, Sandra D. Bakker, Aart van Bochove, Ruud J. L. F. Loffeld

**Affiliations:** Department of Internal Medicine, Zaans Medisch Centrum, 1015 ZW Zaandam, Netherlands

## Abstract

Patients with stage 2 and stage 3 colon cancer often are treated with adjuvant chemotherapy. However, patients seen in daily practice have more comorbidity than those enrolled in clinical trials. This study aims to evaluate prognostic factors for recurrence and to ascertain the benefit of adjuvant chemotherapy on recurrence-free survival (RFS) of patients in a nonselected population. Furthermore, the impact of relative dose intensity (RDI) of adjuvant therapy on RFS is examined. Chart review was performed for 243 consecutive patients diagnosed and treated at a single center for stage 2 and stage 3 colon cancer from 2002 to 2008. Adjuvant chemotherapy was administered to 66 patients. Median overall survival (OS) was 5.84 years and median RFS was 5.37 years. For stage 2 disease, patients treated with or without adjuvant therapy had a median RFS of 5.49 and 5.73, respectively (*p* = ns). For stage 3 disease, median RFS rates were 5.08 and 1.19, respectively (*p* = 0.084). Overall RDI of oxaliplatin based chemotherapy higher than median was associated with increased RFS (*p* = 0.045). In conclusion, adjuvant therapy did not significantly increase recurrence-free survival. This could be the result of comorbidity in patients. Relative dose intensity of oxaliplatin based therapy is associated with RFS.

## 1. Introduction

Colorectal cancer is the third most occurring cancer with an incidence of 80.0 per 100.000 in the year 2011 in Netherlands. Although the prognosis of colon carcinoma has improved significantly over the past years [1], the mortality rate was still 30.5 per 100.000 deaths in 2011, which makes up for 11.8% of total cancer deaths [2].

Curative therapy for colon cancer is largely determined by the lymph node status since positive lymph nodes provide an indication for adjuvant treatment with chemotherapy [3, 4]. Currently the combination of a 5-fluorouracil (5-FU) analogue and oxaliplatin is the treatment of choice [5–7].

Trials treating patients with stage 2 disease with adjuvant therapy show mixed results. A number of studies comparing treatment with fluorouracil/leucovorin (5FU/LV) and observation showed little to no added benefit [8–10]. More recently, studies have been published showing benefit in treating patients with stage 2 disease with an increased risk of recurrence [11–13]. The presence of microsatellite instability (MSI) has been found to decrease the risk of recurrence and negate the effect of adjuvant chemotherapy on RFS in patients with stage 2 disease [14, 15]. The guideline published by the American Society of Clinical Oncology advises against the use of adjuvant therapy with the exception for patients with characteristics that increase risk of recurrence [16]. Patients with MSI and stage 2 colon cancer have no indication for adjuvant therapy.

Studies examining the influence of relative dose intensity (RDI) of adjuvant therapy on RFS in patients with colon cancer treated with 5FU/LV showed no effect of increased duration of therapy on recurrence-free survival (RFS) [17, 18]. However, the effect of RDI on recurrence-free survival in patients treated with adjuvant oxaliplatin based therapy is still relatively unexplored. This information could prove valuable to clinicians and patients because the majority of patients treated with oxaliplatin face unacceptable toxicity resulting in dose reductions, delays, and early termination of treatment leading to a median RDI of 70–85% [19–21].

Published randomized clinical trials poorly represent the day-to-day population treated by clinicians because of major selection and investigator bias [22]. Patients presenting with colon cancer often fulfill the exclusion criteria used in the trials. As such, clinicians have to base treatment decisions on guidelines representing at best only part of their patient population. Previous observational studies show a survival benefit for adjuvant chemotherapy in elderly patients. However, due to their observational nature, these studies are also subject to significant selection bias, only partially corrected through propensity scoring [23, 24].

Therefore, a study was done to evaluate which factors are associated with an increased risk of disease recurrence in patients with stage 2 and stage 3 colon cancer in a nonselected population seen in daily practice. In addition, the effect of adjuvant therapy, and its RDI, on RFS was studied. Subanalyses for the RDI in different regimens were performed.

## 2. Methods

A review of pathology, radiology, and endoscopy reports as well as other correspondence was done for all consecutive patients diagnosed and treated for colorectal cancer at the “Zaans Medisch Centrum,” the community hospital of the Zaanstreek region in the Netherlands, from 2002 to 2008. Evaluation was done on 1-1-2014. In addition, the database of the hospital pharmacy was searched for all prescribed chemotherapy administered in the in- and out-patient clinic. Information on oral medication (capecitabine) was obtained through chart review.

The relative dose intensity of the chemotherapy regimen was measured by averaging the RDI of each individual drug except for leucovorin. The RDI for each drug was calculated by multiplying the time index, the time allotted for the administered chemotherapy cycles divided by the duration of said cycles, and the dose index, the administered cumulative dose divided by the standard cumulative dose. (For the regimens used as reference, see Appendix B [25, 26].)

Relative dose intensity of chemotherapy was dichotomized by dividing patients into groups based on a RDI higher or lower than the median. Associations between RDI and RFS were determined for patients treated with regimens with and without oxaliplatin.

Recurrence-free survival was calculated from date of surgery to date of radiological or histological signs of recurrence. Overall survival was measured from date of diagnosis to date of death.

Patient comorbidity was measured using a Charlson age comorbidity index [27–29].

A full listing of exclusions and detailed description of study variables are noted in the appendices.

Recurrence-free survival outcomes were tested using a Kaplan-Meier analysis. A Log-rank test was used to compare outcomes between groups. Univariate cox regression analysis was used to determine factors associated with increased recurrence-free survival. Patients were censored at death if they had not experienced recurrence. Fisher's exact test and the independent sample *t*-test were used to evaluate differences between patient groups.

Statistical analyses were performed using IBM SPSS statistics software version 20.0 and Microsoft Office Excel 2010.

## 3. Results

**Figure 1:**
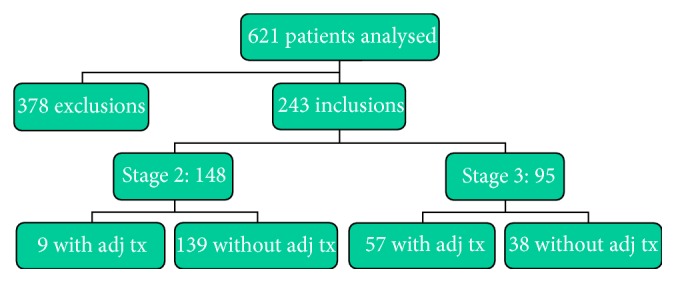
Included patients.

Data were studied of 621 consecutive patients with colorectal cancer treated at the Zaans Medisch Centrum. Three hundred seventy-eight patients were excluded for the present analysis (see Appendix A). One hundred forty-three patients were diagnosed with rectal cancer, 149 patients presented with stage 0, 1, or 4 colon cancer, and 78 patients were excluded for other reasons. In this analysis 243 patients, 95 with stage 3 and 148 with stage 2, were included (Figure 1). Four patients with stage 2 disease could not be included in the cox regression analyses since they died almost immediately after surgery as a result of perioperative complications; hence, there was insufficient survival time.

All patients were followed for at least 5 years, or until death of any cause (range 0.0–11.8). Median follow-up of patients was 5.84 years, interquartile range (IQR) 3.00–7.84. Disease recurrence occurred in 68 patients (28%): 29 patients (20%) with stage 2 disease and 39 patients (41%) with stage 3 disease (Table 1).

In patients with stage 2 disease the number of examined lymph nodes was inversely related to the risk of recurrence with a hazard ratio (HR) of 0.92 per node examined (Table 1).

The following variables in patients with stage 3 disease were associated with recurrence-free survival: N-stage (HR = 0.32 for N1 versus N2), number of metastatic lymph nodes (HR 1.14 per positive node), LNR (HR 11.64 per point increase), tumor site (HR 0.47 for distal versus proximal tumors), and lymph vascular or perineural invasion (LVI or PNI) (HR = 0.43 for patients without LVI/PNI).

Nine patients (6%) with stage 2 and 57 patients (60%) with stage 3 disease received adjuvant chemotherapy consisting of either a 5-fluorouracil analogue, or folfox/capox (regimens consisting of either 5-fluorouracil and leucovorin or capecitabine in combination with oxaliplatin). There was no significant improvement in RFS when patients were treated with adjuvant chemotherapy. However, patients with stage 3 disease treated with adjuvant therapy did display a trend towards improvement with a 3.89-year longer median RFS.

**Figure 2:**
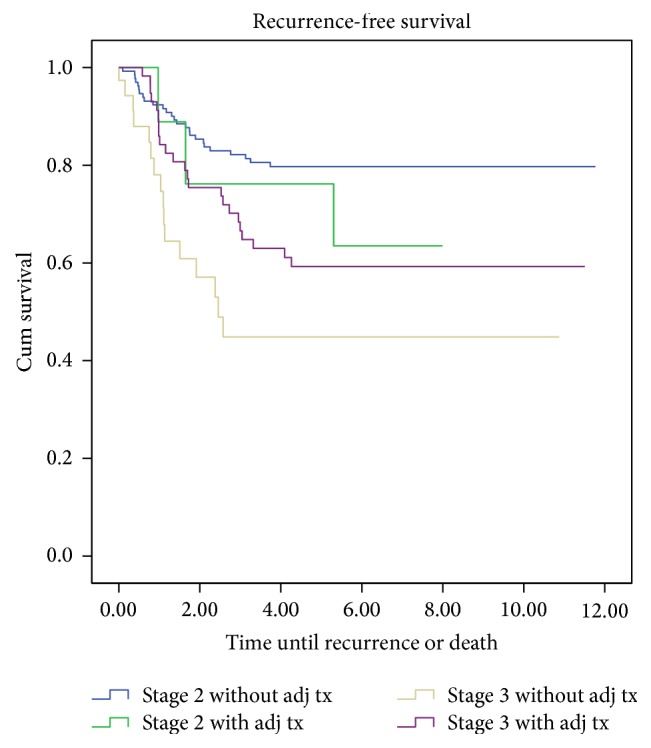
Kaplan Meier analysis of recurrence-free survival of patients with or without adjuvant therapy.

With almost identical recurrence rates (*p* = ns), this trend is the result of the significantly longer overall survival in the stage 3 patients treated with chemotherapy (*p* < 0.001). Patients with stage 3 disease without adjuvant treatment had significantly more comorbidity according to the Charlson index (*p* < 0.001). Therefore, they had a shorter life expectancy based on age and preexistent conditions. This reduces the relative risk of death from tumor progression (Table 2 and Figure 2).

**Figure 3:**
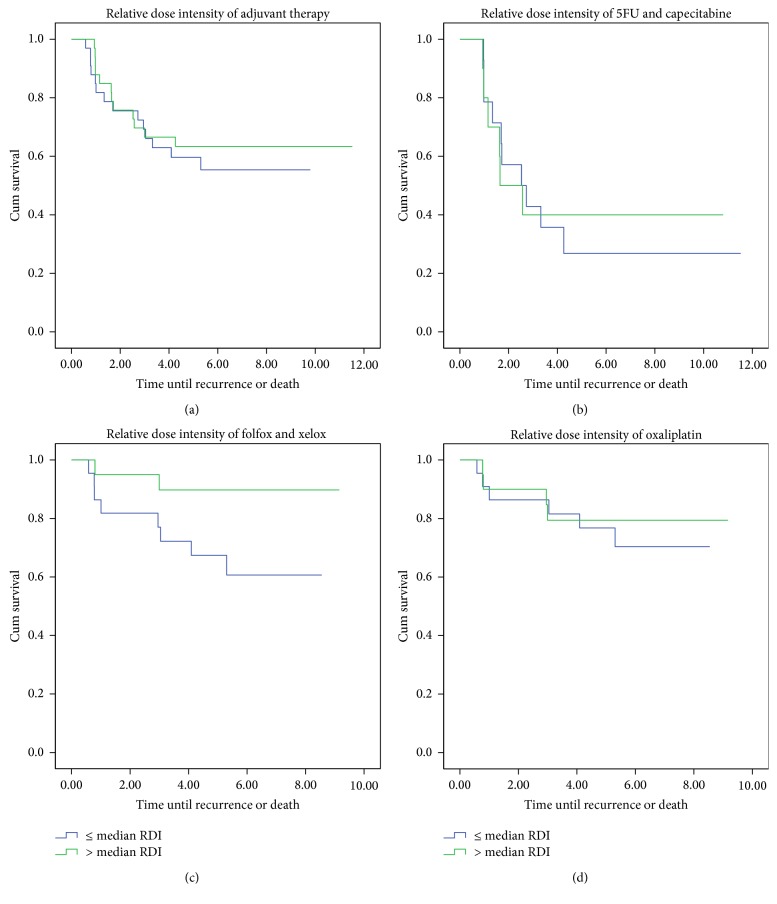
Kaplan Meier analyses of recurrence-free survival based on relative dose intensity (RDI) of adjuvant chemotherapy.

Patients receiving adjuvant treatment with folfox or capox with a RDI higher than the median showed significant improvement of RFS (*p* = 0.04). However, the subanalysis of the oxaliplatin dose intensity in patients treated with folfox or capox showed no significant improvement in RFS (Table 3 and Figure 3).

## 4. Discussion

This study deals with treatment of colon cancer in daily practice. Eighteen patients (3%) were referred to a specialized cancer center, either at their own request or for treatment not available in this center at this time, for example, partial hepatectomy. This introduces some inevitable selection bias. The long inclusion period of this cohort inadvertently causes differences in adjuvant treatment between patients diagnosed in 2002 versus 2008, the most important of which is the addition of oxaliplatin to adjuvant therapy in 2004. In this cohort 81% of patients treated with oxaliplatin based therapy received a folfox regimen. In many centers the preferred treatment is capox therapy. While capox is associated with a lower RDI, no significant difference in OS and RFS has been observed between treatments [30].

Overall survival in this cohort is underestimated in patients not treated with adjuvant therapy compared to patients treated with adjuvant therapy and those observed in other cohorts due to the fact that patients dying of perioperative complications are included in this analysis. (See Appendix D for characteristics of these patients.) Since most of these patients are octogenarians and have a high Charlson index it seems reasonable to include them in the group not treated without adjuvant therapy as most would not qualify regardless.

This study shows an inverse correlation between the number of lymph nodes examined and the risk of recurrence in patients with stage 2 disease and a trend towards increased risk of recurrence for patients with poorly differentiated tumors, LVI or PNI, and T4 status. Similar results were obtained for patients with stage 3 disease except for a significantly increased risk of recurrence for proximal tumors and an increased LNR or N2 status. These results are in line with previous reports except for the association between tumor site and RFS [12, 16, 31–36]. As such, more evidence is needed to support this observation.

Several trials and meta-analyses have been performed to evaluate the added benefit of adjuvant chemotherapy in patients with stage 2 colon cancer. Many studies in the past have been insufficiently powered, and most of the evidence has come from pooled meta-analyses of trials including both patients with stage 2 and stage 3 disease [8–11, 16, 37, 38]. Currently, only patients with stage 2 disease who are perceived to be at an increased risk of recurrence and without microsatellite instability have an indication for adjuvant treatment. This reflects the treatment strategy in Netherlands and might explain the observation of a, albeit not significant, higher recurrence rate in stage 2 patients treated with adjuvant therapy. Although this study contains only 9 patients with stage 2 disease treated with adjuvant therapy, this suggests that these patients are at increased risk of recurrence and might benefit from adjuvant treatment. This hypothesis is confirmed by previous findings from other studies [12, 13, 39–42] and supports the policies outlined in the current Dutch and American guidelines for adjuvant treatment of colon cancer [16, 43].

In this cohort, patients with stage 3 disease treated with adjuvant therapy experienced a nonsignificant increase in recurrence-free survival compared to those treated with surgery alone. Recurrence rates were almost identical in patients treated with or without adjuvant chemotherapy. Since a significant survival benefit of adjuvant therapy in stage 3 disease has been demonstrated in multiple large randomized trials, the results observed here are somewhat disappointing [3–5, 41]. This could be due to a combination of lack of statistical power and a small effect size. This effect reduction can be explained by differences in characteristics between patients treated in daily life or in controlled clinical trials. Comparing the present population to that of the MOSAIC and the NO16968 trial, the median age in daily life is approximately 10 years higher. The median dose intensity of oxaliplatin was 11–13% lower in this cohort. The dose intensity of 5-FU single agent therapy was similar, although the MOSAIC study only describes a maximum dose index in 87% of patients. Furthermore, these trials have stricter exclusion criteria with regard to comorbidity such as the NO16968 trial requiring an ECOG performance score of 1 or 0 and a life expectancy of at least five years [6, 19]. As such, one can conclude that the results from these trials might overestimate the benefit of adjuvant treatment and cannot be extrapolated to a majority of patients presenting with stage 3 disease in normal daily practice.

The similar recurrence rates observed in this cohort in patients treated with or without adjuvant chemotherapy indicate that increased comorbidity and reduced overall survival decrease the efficacy of adjuvant therapy as they increase the risk of death from nontumor related events. Thus a patient's survival benefit from adjuvant therapy is directly related to his or her life expectancy and should play an important role in the treatment decisions made by patient and clinician.

Regardless of the potential survival benefit, the toxicity and adverse events caused by adjuvant chemotherapy, especially oxaliplatin, result in significant patient morbidity [20, 21]. The notion that higher doses of chemotherapy, if tolerated, improve cancer related survival seems obvious, yet randomized controlled trials evaluating increased doses of chemotherapy show mixed results [44–47]. Chau et al. observed noninferiority of a three-month treatment schedule with 5FU/LV instead of six, and the GERCOR study showed no effect of longer treatment with 5FU/LV [17, 18].

Although this retrospective analysis of the effect of dose intensity of chemotherapy on survival introduces bias based on comorbidity and treatment strategy, most bias was removed by evaluating recurrence-free survival in an adjuvant setting. A significant association between the RDI of oxaliplatin based therapy and recurrence-free survival was observed. This did not translate into an effect of the isolated oxaliplatin dose on RFS and as such seems to be mostly dependent on the RDI of 5-FU analogues. This could create an opportunity to lower the dose of oxaliplatin and reduce invalidating polyneuropathy without significantly impacting outcomes. However, clinicians should proceed cautiously as these results do indicate an effect of dose intensity on outcomes in adjuvant treatment of colon cancer. Judgment should be withheld until results from a larger prospective study are presented.

In conclusion, this study presents evidence that the effect of adjuvant chemotherapy is overestimated in previously reported randomized clinical trials and does not reflect a nonselected population since comorbidity is not factored into the equation. Furthermore, a high relative dose intensity of oxaliplatin based adjuvant therapy is associated with improved recurrence-free survival. Counseling the heterogeneous group of patients with stage 2 and 3 colon cancer about the benefits and downsides of (continuing) adjuvant therapy should be performed on a case by case basis.

## What Does This Paper Add to the Literature?

Colon cancer is a malignancy occurring mostly in older patients. Results of adjuvant chemotherapy are based on younger, usually fit patients. Older patients often have comorbidity rendering results of adjuvant therapy disappointing. Clinicians should take comorbidity and life expectancy into account when deciding to give adjuvant chemotherapy.

## Figures and Tables

**Table 1:** Patient characteristics and univariate cox regression analysis of effect on recurrence-free survival.

	Stage 2	Cox HR for recurrence	95% CI	Stage 3	Cox HR for recurrence	95% CI	Total
Gender (%)							
Male	78 (53)			46 (48)			124 (51)
Female	70 (47)			49 (52)			119 (49)
Age, median (iqr)	73,5 (63,7–80,0)			69,3 (62,9–76,7)			72,2 (63,2–79,5)
T-stage (%)							
3	123 (83)	0,64	0,27–1,49	66 (69)	0,53	0,27–1,01	189 (78)
4	25 (17)			25 (26)			50 (21)
Tumor site (%)							
Distal	62 (42)	1,1	0,52–2,33	48 (51)	0,47	0,24–0,91	110 (45)
Proximal	77 (52)			44 (46)			121 (50)
Poor differentiation (%)	13 (9)	na		25 (26)	1,89	0,98–3,70	38 (16)
LVI or PNI (%)	15 (10)	1,89	0,72–5,00	17 (18)	2,33	1,12–4,76	32 (13)
N-stage (%)							
0	148 (100)			0			148 (61)
1	0			61 (64)	0,32	0,17–0,60	61 (25)
2	0			34 (36)			34 (14)
Median # LN examined (iqr)^*∗*^	13 (8–19)	0,92	0,87–0,98	13 (8–19)	0,97	0,93–1,01	13 (8–19)
Median # metastatic LN (iqr)^*∗*^	0			2 (1–6)	1,14	1,08–1,21	0 (0–2)
Median LNR (iqr)^*∗*^	0			0,29 (0,11–0,50)	11,64	4,10–32,99	0 (0–0,20)
Adjuvant therapy (%)	9 (6)	1,81	0,55–5,88	57 (60)	0,57	0,30–1,09	66 (27)
Recurrence (%)	29 (20)			39 (41)			68 (28)
Median OS (iqr)	6,38 (4,25–8,49)			5,00 (1,84–7,09)			5,84 (3,00–7,84)
Median RFS (iqr)	5,72 (2,38–7,97)			3,04 (1,01–7,04)			5,37 (1,65–7,58)
Total	**148**			**95**			**243**

HR = hazard ratio; CI = confidence interval; iqr = interquartile range; LVI = lymphovascular invasion; PNI = perineural invasion; LN = lymph node; LNR = lymph node ratio; OS = overall survival; RFS = recurrence-free survival.

^*∗*^For continuous variables, the hazard ratio is expressed per point increase.

**Table 2:** Effect of adjuvant therapy on (recurrence-free) survival.

	Stage 2 − adj Tx	Stage 2 + adj Tx	*p*	Stage 3 − adj Tx	Stage 3 + adj Tx	*p*
Number of patients	139	9		38	57	
Recurrence rate	0,19	0,33	0,379	0,42	0,4	1,000
Median OS (iqr)	6,42 (4,25–8,59)	6,17 (2,08–7,42)	0,772	1,79 (0,60–6,07)	5,51 (4,06–7,47)	0,000
Median RFS (iqr)	5,73 (2.76–8,11)	5,49 (1.33–7,31)	0,325	1,19 (0,36–5,94)	5,08 (2,12–7,40)	0,084
Median age (iqr)	75,3 (65,4–80,4)	61,0 (59,3–65,7)	0,002	78,7 (73,9–83,1)	63,5 (59,8–69,8)	0,000
Median Charlson index (iqr)	5 (3–6)	3 (2–4.5)	0,038	5 (4–6)	3 (3-4)	0,000
Cause of death (%)						
Alive	87 (56)	5 (56)		8 (21)	32 (56)	
Cancer	18 (13)	3 (33)		15 (40)	18 (32)	
Treatment	3 (2)	1 (11)		8 (21)	0	
Other	29 (21)	0		6 (16)	5 (9)	
Unknown	11 (8)	0		1 (3)	2 (4)	

Adj Tx = adjuvant therapy; iqr = interquartile range; OS = overall survival; RFS = recurrence-free survival.

**Table 3:** Influence of relative dose intensity on recurrence-free survival.

	> median RDI	≤ median RDI	*p*	Total
Total				
Number of pts	33	33		66
Median RDI (iqr)	0,92 (0,86–0,98)	0,65 (0,30–0,76)	0,000	0,83 (0,64–0,92)
Recurrence rate	0,36	0,42	0,801	0,39
Median RFS (iqr)	5,49 (2,12–7,91)	5,08 (1,52–6,83)	0,590	5,22 (1,71–7,39)
Folfox/capox				
Number of pts	20	22		42
Median RDI (iqr)	0,89 (0,84–0,95)	0,71 (0,44–0,76)	0,000	0,82 (0,70–0,87)
Recurrence rate	0,1	0,36	0,071	0,24
Median RFS (iqr)	6,94 (5,32–7,65)	5,11 (2,47–6,93)	0,045	5,51 (3,83–7,21)
Oxaliplatin^*∗*^				
Number of pts	20	22		42
Median RDI (iqr)	0,88 (0,74–0,95)	0,50 (0,39–0,63)	0,000	0,67 (0,50–0,87)
Recurrence rate	0,2	0,27	0,732	0,24
Median RFS (iqr)	6,94 (3,40–7,65)	5,36 (3,83–6,93)	0,602	5,51 (3,83–7,21)
5FU				
Number of pts	10	14		24
Median RDI (iqr)	1,00 (0,94–1,00)	0,55 (0,26–0,87)	0,000	0,87 (0,36–0,99)
Recurrence rate	0,6	0,71	0,673	0,67
Median RFS (iqr)	2,11 (1,11–10,21)	2,63 (1,25–5,50)	0,865	2,55 (1,20–9,47)

RDI = relative dose intensity; pts = patients; iqr = interquartile range; RFS = recurrence-free survival; 5FU = 5-fluorouracil (also includes capecitabine).

^*∗*^Subanalysis of oxaliplatin RDI in patients treated with capox or folfox.

**Table 4:** 

Exclusions:	Number 378
Patients with stage 0 colon cancer	7
Patients with stage 1 colon cancer	44
Patients with stage 4 colon cancer	92
Patients with colon cancer of unknown disease stage	6
Patients with rectum cancer	143
Other exclusions	86
Benign pathology:	16
Referred for treatment in other hospitals:	18
Missing data:	20
Patient with incorrect information	5
Endoscopically removed carcinoma in situ	4
Recurrence of earlier colon cancer	10
Nonadenocarcinoma of the colon	13
Urothelial cell carcinoma	3
Rhabdomyosarcoma	1
Lung cancer	1
Non-Hodgkin lymphoma	1
Ovarial cancer	1
Pancreatic cancer	1
Breast cancer	1
Anal cancer	1
Carcinoid of the colon	2
Neuroendocrine tumor of the colon	1

**Table 5:** 

Points	1	2	3	6
Morbidity	MI	Hemiplegia	Moderate-severe liver disease	Metastatic solid tumour
	CCF	Moderate-severe		
	PVD	CRF		AIDS
	COPD	DM (with end-organ damage)		
	DM (without end-organ damage)	Malignancy		
	Cerebrovascular disease	Leukaemia		
	Dementia	Lymphoma		
	Ulcers			
	Connective tissue disease			
	Mild liver disease			

MI, myocardial infarction; CCF, congestive cardiac failure; PVD, peripheral vascular disease; COPD, chronic obstructive pulmonary disease; DM, diadetes mellitus; CRF, chronic renal failure.

**Table 6:** 

Case #	Gender	Age	Disease stage	Cause of death	Charlson index
1	Female	84	3	Anastomotic leak	5
2	Female	83	3	Anastomotic leak	8
3	Male	79	3	Abdominal septicemia, multiorgan failure	5
4	Female	82	3	Anastomotic leak	6
5	Female	88	3	Anastomotic leak	5
6	Male	75	3	Mesenteric thrombosis, bowel perforation	4
7	Male	69	3	Spinal bleeding, abdominal septicemia, and multiorgan failure	7
8	Male	85	2	Rebleeding after surgery	7
9	Male	67	2	Adrenal insufficiency, septicemia	5
10	Female	88	2	Pneumonia, bowel obstruction	5

## Appendices

### A. Exclusions

See Table 4.

### B. Definition of Variables

*Overall Survival*. Time of diagnosis (per month) until time of death (per month).

*Recurrence-Free Survival*. Date of resection until date of recurrence (radiologically or pathologically confirmed), censored at date of death.

TNM classification according to TNM7 classification as deduced from pathology report: T-stage 1 and T-stage 2 were excluded in assessment of risk of recurrence of T4 tumors in patients with stage 3 disease.

*Tumor Differentiation*. Poor and poor to moderate versus moderate, moderate to good, and good.

Lymphovascular and/or perineural invasion is as described by pathologist in the pathology report.

*Lymph Node Ratio*. Number of metastatic lymph nodes, divided by number of examined lymph nodes.

*Tumor Site*. Distal colon consists of descending and sigmoid colon. The proximal colon is defined as the part that lies proximal to the splenic flexure. Synchronous tumors were excluded from analyses regarding tumor site.

*Relative Dose Intensity per Therapeutic Agent*. Dose index, the percentage of cumulative dose administered divided by planned cumulative dose. Changes to dose smaller than 20% were assumed to be the result of weight change. Time index was calculated by dividing the time allotted for the administered treatment cycles divided by the actual duration until completion of said cycles. Relative dose intensity was calculated by multiplying dose index and time index.

The chemotherapy regimens used as reference were the following:

*5-FU + LV (Roswell Park Regimen)*

5-FU 500 mg/m^2^ iv bolus 1 h after the start of leucovorin,
Leucovorin 500 mg/m^2^ iv over 2 hrs,
Qw × 6 wks every 8 wks for 3-4 cycles.

*5-FU + LV (Mayo Clinic Regimen)*

5-FU 370–425 mg/m^2^/d iv bolus d1–5,
Leucovorin 20–25 mg/m^2^/d iv bolus d1–5,
Q4w × 6 cycles.

*Capecitabine*

Capecitabine (Xeloda) 1250 mg/m^2^ po bid × 14 days,
Q3w × 8 cycles.

*FOLFOX4*

Leucovorin 200 mg/m^2^ iv over 2 hrs before 5-FU, d1 and 2,
5-FU 400 mg/m^2^ iv bolus and then 600 mg/m^2^ iv over 22 hrs, d1 and d2,
Oxaliplatin (Eloxatin) 85 mg/m^2^ iv d1,
Q2w × 12 cycles.

*CAPOX*

Capecitabine (Xeloda) 1000 mg/m^2^ po bid × 14 days,
Oxaliplatin (Eloxatin) 130 mg/m^2^ iv over 2 hrs d1,
Q3w × 8 cycles.

*Cause of Death.* Defined as categorical variable consisting of the following categories: treatment related, tumor related, other, unknown, and alive. If a patient is referred back to the primary care physician or has no more treatment options for a metastasized malignancy, he or she is assumed to have died from tumor progression. When a patient is lost to follow-up, he or she is assumed to have died from other causes if there is a disease-free interval of at least 5 years.

### C. Charlson Age Comorbidity Index

The Charlson index was measured at time of diagnosis. The diagnosed colon cancer was not taken into account when calculating the total score (see Table 5).

*Comorbidity Score*. Add up the corresponding amount of points for each condition present.

*Age Score*. [Patient age]/10 − 4, always rounded up.

*Charlson Index*. Sum of comorbidity and age score.

### D. Characteristics of Patients Dying from Perioperative Complications of Primary Surgery

See Table 6.
